# Taxono-genomics and description of *Gordonibacter massiliensis* sp. nov., a new bacterium isolated from stool of healthy patient

**DOI:** 10.1016/j.nmni.2019.100624

**Published:** 2019-11-29

**Authors:** I.I. Ngom, I. Hasni, C.I. Lo, S.I. Traore, A. Fontanini, D. Raoult, F. Fenollar

**Affiliations:** 1) Aix Marseille Université, IRD, AP-HM, MEФI, Marseille, France; 2) IHU-Méditerranée Infection, Marseille, France; 3) Aix Marseille Université, IRD, AP-HM, SSA, VITROME, Marseille, France

**Keywords:** Culturomics, *Gordonibacter massiliensis*sp. nov., human gut, stool, taxono-genomics

## Abstract

Using the taxono-genomics concept, we describe here a strictly anaerobic Gram-positive bacillus. This strain was isolated from the stool sample of a 50-year-old healthy Bedouin woman. The 16S rRNA gene sequence analysis and the whole-genome sequencing showed that this isolate belonged to the genus *Gordonibacter* in the family *Eggerthellaceae*. Based on these criteria, we propose the creation of *Gordonibacter massiliensis* sp. nov., strain Marseille-P2775^T^ (= CSUR P2775).

## Introduction

Members of the genus *Gordonibacter* are Gram-positive bacteria belonging to the recent family *Eggerthellaceae* [1]. These species are part of the human gut microbiota and have the capacity to metabolize polyphenols from diet into bioavailable metabolites known as urolithin [2,3]. During the last decades, culturomics studies have brought insight into the human microbiota, which has led to the discovery of previously uncultured bacteria [4,5]. Culturomics, including different culture conditions, is completed by matrix-assisted laser desorption/ionization time-of-flight (MALDI-TOF) identification and sequencing of the 16S rRNA gene, in order to explore the microbial diversity of the human gut [6,7]. This new bacterial species was described using a combination of genotypic and phenotypic characteristics according to the previously reported taxono-genomics approach [8,9].

Herein, we give details of the isolation and taxono-genomics characters of strain Marseille-P2775^T^, which is the type strain of *Gordonibacter massiliensis* sp. nov.

## Isolation and growth conditions

**Fig. 1:**
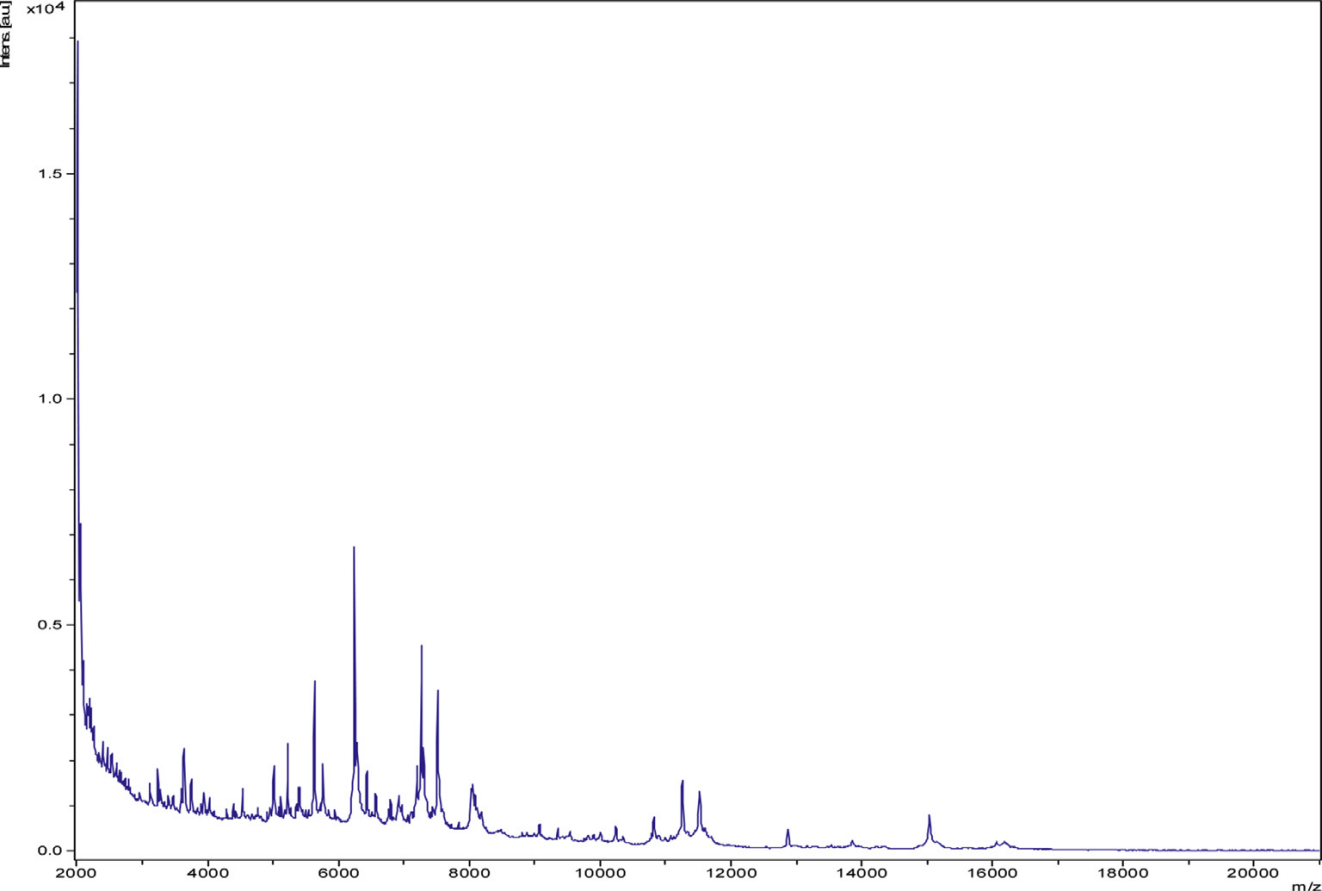
MALDI-TOF MS reference spectrum generated from the Biotyper 3.0 software. Spectra from 12 individual colonies were compared with the aim to obtain consensual spectrum.

The strain was isolated in 2016 from the stool sample of a 50-year-old healthy Bedouin woman living in the Jazan region of Saudi Arabia. This study was performed in Franceafter approval from the ethics committee of the King Abdulaziz University (Saudi Arabia) and the local ethics committee of the IFR48 (Marseille, France) under numbers 014-CEGMR-2-ETH-P and 09-022, respectively. Isolation and growth conditions of strain were performed as previously described [10]. The initial growth of strain Marseille-P2775 was obtained after 2 days of incubation in a Colombia agar supplemented with 5% sheep's blood (COS, bioMérieux, Marcy l’Étoile, France) under strict anaerobic conditions at 37°C. Identification of this bacterial strain was attempted using MALDI-TOF mass spectrometry. The screening was performed on a Microflex LT spectrometer (Bruker Daltonics, Bremen, Germany) as previously reported [11]. The spectra obtained were saved into MALDI Biotyper 3.0 software (Bruker Daltonics) and analysed against the main spectra of the bacteria included in the local URMS database (https://www.mediterranee-infection.com/urms-data-base) (Fig. 1).

## Strain identification

**Fig. 2:**
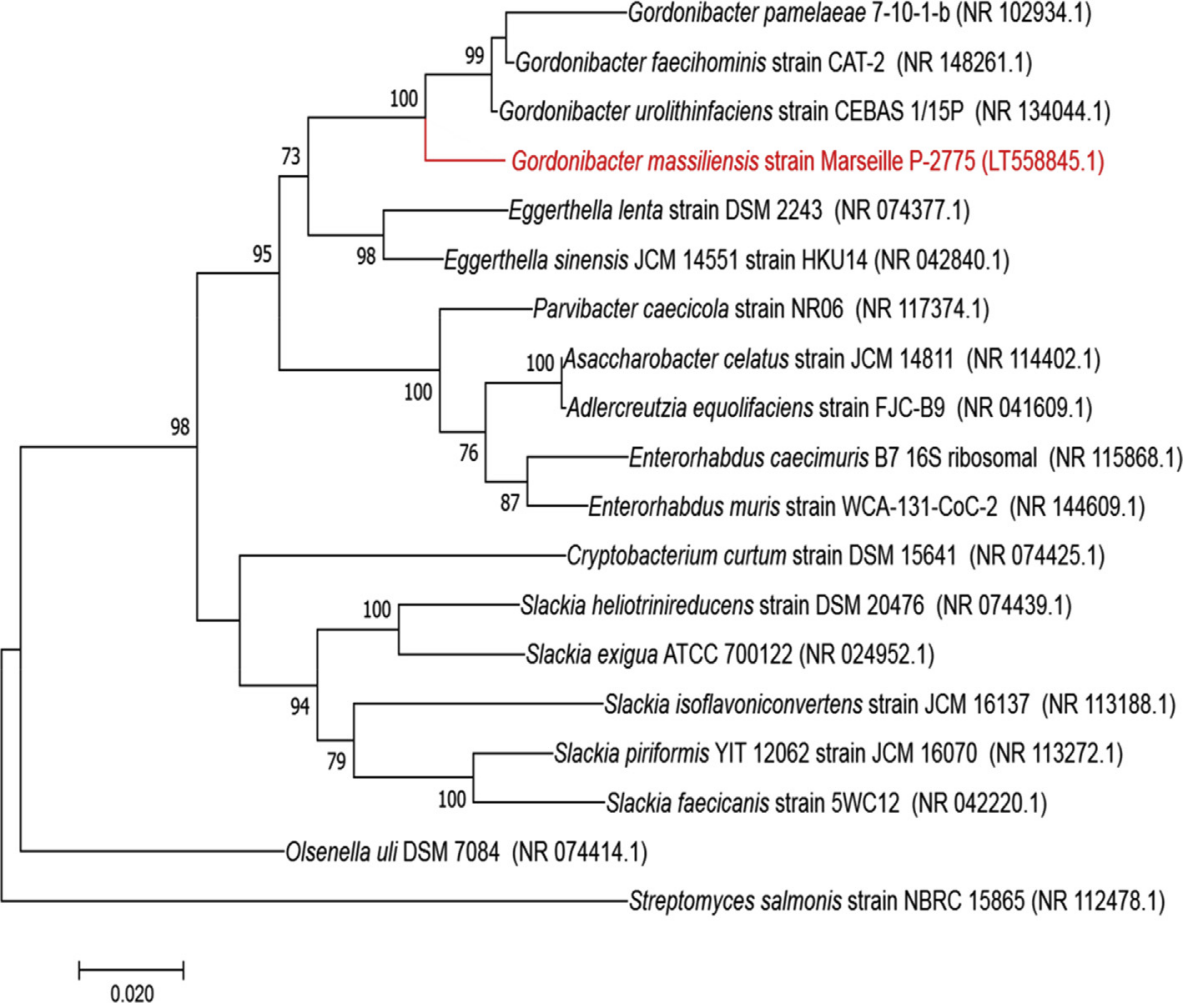
Phylogenetic tree showing the position of *Gordonibacter massiliensis* strain Marseille-P2775^T^ and other phylogenetically close neighbours. The respective GenBank accession numbers for 16S rRNA genes are indicated in parenthesis. Sequences were aligned using Muscle v7.0.26 with default parameters and phylogenetic inferences were obtained using the maximum likelihood method with MEGA 7 software. Numbers at the nodes are percentages of bootstrap values obtained by repeating the analysis 1000 times to generate a majority consensus tree. Only bootstrap values > 70% were retained. The scale bar indicates a 5% nucleotide sequence divergence.

To classify the strain Marseille-P2775, its 16S rRNA gene was amplified using the fD1 and rP2 primer pair (Eurogentec, Angers, France) and sequenced using the Big Dye® Terminator v1.1 Cycle Sequencing Kit and 3500xLGenetic Analyser capillary sequencer (Thermofisher, Saint-Aubin, France) [12,13] as described previously. The 16S rRNA nucleotide sequences were assembled and corrected using CodonCode Aligner software (http://www.codoncode.com). The 16S rRNA gene sequence analysis of strain Marseille-P2775 showed 97.19% identity with *Gordonibacter urolithinfaciens* strain CEBAS 1/15P (GenBank accession number: NR134044), the phylogenetically closest species with a standing in nomenclature (Fig. 2). This value was <98.7% of similarity, the threshold above which a strain is considered a new species [14].

## Phenotypic characteristics

**Fig. 3:**
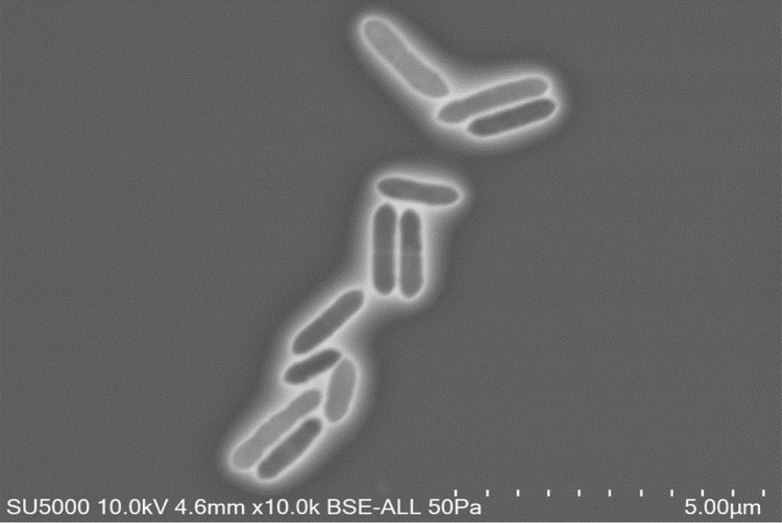
Scanning electron micrograph of *Gordonibacter massiliensis* strain Marseille-P2775^T^ obtained from TM4000 microscope. Scale bar and acquisition settings are shown on the picture.

Growing on Columbia blood agar, colonies of strain Marseille-P2775 appeared beige with a mean diameter of 1 mm. Bacterial cells were Gram-positive, strictly anaerobic short-rod bacilli with a mean length of 1.2 μm and 0.5 μm in diameter (Fig. 3). Strain Marseille-P2775 was motile, non-haemolytic and non-spore-forming. It presented catalase-positive and oxidase-negative activities. Carbohydrate metabolism and enzymatic characteristics of the strain Marseille-P2775 were tested under strict anaerobic conditions at 37°C using API 50 CH and ZYM, respectively (Table 1). A comparative study of the differential characteristics of this strain with other closely related species is shown in Table 2. Cellular fatty acid methyl ester (FAME) analysis was performed as previously described [15,16]. The major fatty acids were 9-octadecenoic acid (41%) and hexadecanoic acid (24%). Several branched structures were also described with lower abundances (Table 3).

**Table 1:** Biochemical tests performed on strain Marseille-P2775 using API strips 50 CH and ZYM

API 50 CH				API ZYM	
Tests	Results	Tests	Results	Tests	Results
Control	−	Esculin ferric citrate	+	Control	−
Glycerol	+	Salicin	+	Alkaline phosphatase	−
Erythritol	−	d-cellobiose	−	Esterase (C4)	+
d-arabinose	−	d-maltose	+	Esterase lipase (C8)	+
l-arabinose	+	d-lactose	+	Lipase (C14)	−
d-ribose	−	d-melibiose	−	Leucine arylamidase	+
d-xylose	+	d-saccharose	+	Valine arylamidase	−
l-xylose	−	d-trehalose	+	Cystine arylamidase	+
d-adonitol	−	Inulin	−	Trypsin	−
Methyl β-d-xylopyranoside	−	d-melezitose	+	α-chymotrypsin	−
d-galactose	+	d-raffinose	−	Acid phosphatase	+
d-glucose	+	Amidon	−	Naphthol-AS-BI-phosphohydrolase	+
d-fructose	+	Glycogen	−	α-galactosidase	−
d-mannose	−	Xylitol	−	β-galactosidase	+
l-sorbose	−	Gentiobiose	+	β-glucuronidase	−
l-rhamnose	−	d-tyranose	+	α-glucosidase	−
Dulcitol	−	d-lyxose	−	β-glucosidase	−
Inositol	−	d-tagatose	−	*N*-acetyl-β-glucosaminidase	+
d-mannitol	+	d-fucose	−	α-mannosidase	−
d-sorbitol	+	l-fucose	−	α-fucosidase	−
Methyl α-d-mannopyranoside	−	d-arabitol	−		
Methyl α-d-glucopyranoside	−	l-arabitol	−		
*N*-acetyl-glucosamine	+	Potassium gluconate	−		
Amygdalin	−	Potassium 2-ketogluconate	−		
Arbutin	−	Potassium 5-ketogluconate	−

**Table 2:** Differential characteristics of *Gordonibacter massiliensis* strain Marseille-P2775, *Gordonibacter urolithinfaciens*, *Gordonibacter pamelaeae*, *Eggerthella lenta* and *Eggerthella sinensis*

Properties	*Gordonibacter massiliensis*	*Gordonibacter urolithinfaciens*	*Gordonibacter pamelaeae*	*Eggerthella lenta*	*Eggerthella sinensis*
Cell diameter (μm)	0.5	0.4–0.6	0.5–0.6	0.5	0.5
Oxygen requirement	Anaerobic	Anaerobic	Anaerobic	Anaerobic	Anaerobic
Shape	Coccobacilli	Coccobacilli	Coccobacilli	Coccobacilli	Coccobacilli
Gram stain	+	+	+	+	+
Motility	+	+	+	−	−
Sporulation	−	−	−	−	−
Production of:					
Alkaline phosphatase	−	−	−	−	−
Catalase	+	+	+	Variable	+
Oxidase	−	NA	NA	NA	+
β-galactosidase	+	+	NA	−	−
N-acetyl-glucosamine	+	NA	NA	−	−
Acid from:					
l-arabinose	+	−	−	−	−
Mannose	−	−	−	+	−
d-fucose	−	+	NA	NA	NA
l-fucose	−	+	−	−	+
d-glucose	+	−	−	−	−
Trehalose	−	−	−	+	−
d-fructose	+	+	−	NA	NA
G + C content (%)	65.1	66.4	66.4	62	65.6
Habitat	Human gut	Human gut	Human gut	Human gut	Human gut

+, positive result; −, negative result; NA, data not available.

**Table 3:** Cellular fatty acid composition (%) of strain Marseille-P2775^T^ compared with other *Gordonibacter* species

Fatty acids	Name	*Gordonibacter massiliensis*	*Gordonibacter pamelaeae*	*Gordonibacter faecihominis*
12:00	Dodecanoic acid	TR	2.70	ND
13:00	Tridecanoic acid	TR	4.20	ND
13:0 anteiso	10-methyl-Dodecanoic acid	1.0	TR	ND
13:0 iso	11-methyl-Dodecanoic acid	2.3	1.09	ND
14:00	Tetradecanoic acid	8.5	8.71	8.9
14:0 iso	12-methyl-Tridecanoic acid	2.6	6.78	8.4
15:00	Pentadecanoic acid	1.0	TR	ND
15:0 anteiso	12-methyl-tetradecanoic acid	6.6	19.79	8.4
15:0 iso	13-methyl-tetradecanoic acid	1.9	2.96	4.3
16:00	Hexadecanoic acid	23.9	2.42	10.5
16:1n7	9-Hexadecenoic acid	TR	1.69	3.6
17:00	Heptadecanoic acid	TR	0.68	ND
17:01	Heptadecenoic acid	TR	ND	ND
18:00	Octadecanoic acid	7.2	TR	TR
18:1n9	9-Octadecenoic acid	40.8	3.65	ND
18:2n6	9,12-Octadecadienoic acid	1.5	TR	ND

TR, trace amounts <1%; ND, not detected.

## Genome sequencing

**Fig. 4:**
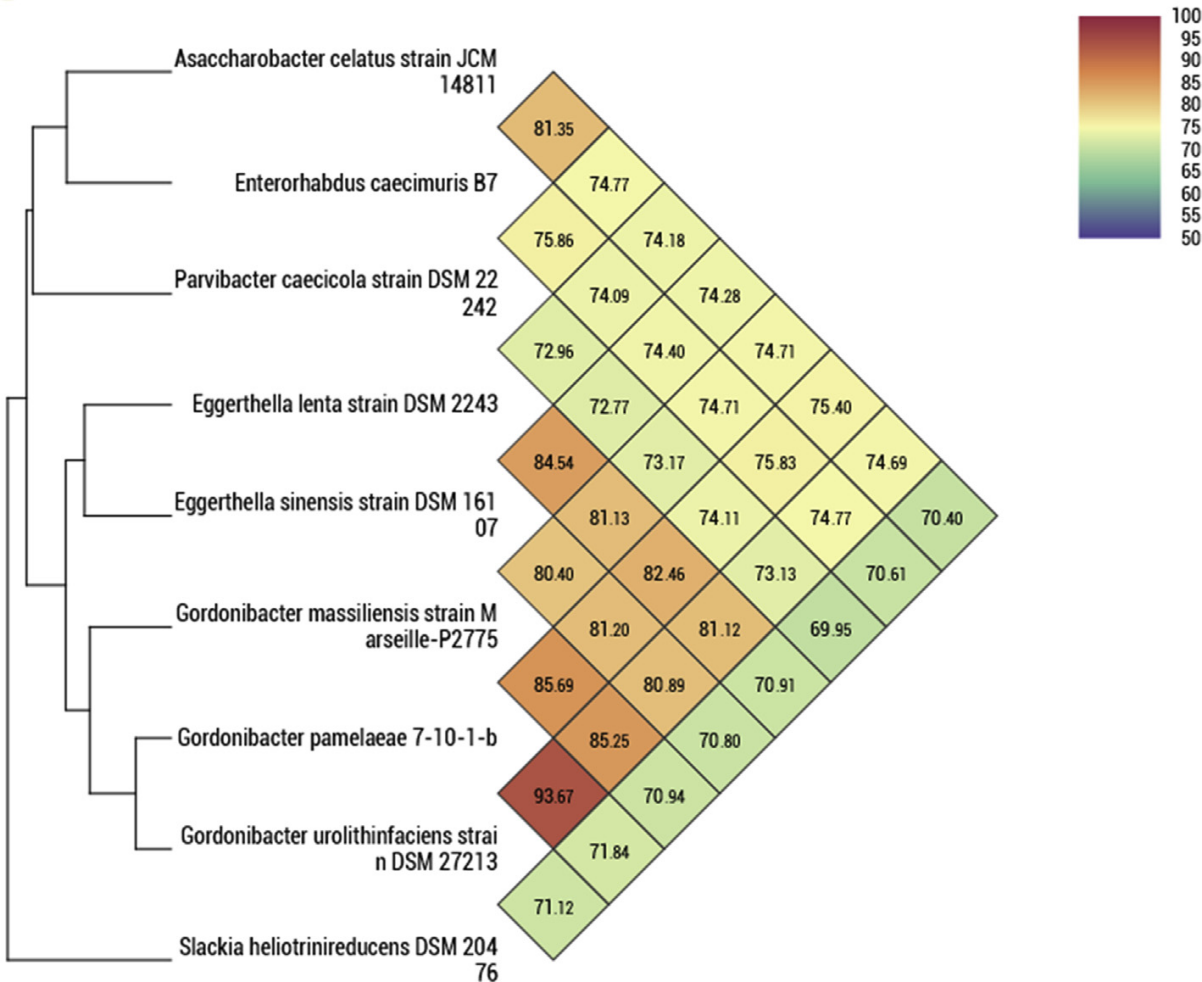
Heatmap generated with OrthoANI values calculated using the OAT software between *Gordonibacter massiliensis* strain Marseille-P2775^T^ and other closely related species with standing in nomenclature.

The DNA genomic extraction was performed using the EZ1 biorobot and the EZ1 DNA tissue kit. Genomic DNA (gDNA) was quantified by a Qubit assay. The sequencing was performed using MiSeq technology (Illumina, San Diego, CA, USA) with the Paired-End (Illumina). The assembly was performed with Spades software [17]. The reads with low quality were trimmed using Trimmomatic software [18]. GapCloser software [19] was used to reduce the assembly gap. Scaffolds < 800 bp and scaffolds with a depth value < 25% of the mean depth were removed. The total length of the *G. massiliensis* genome is 3.9 megabases encompassing 1 contig with a G + C content of 65.1 mol%. The predicted genes analysis reported 3248 genes. The degree of genomic similarity of strain Marseille-P2775 with closely related species was estimated using the OrthoANI software [20]. OrthoANI values ranged from 69.95% between *Parvibacter caecicola* strain DSM 22242 and *Slackia heliotrinireducens* strain DSM 20476 to 93.67% between *Gordonibacter urolithinfaciens* strain DSM 27213 and *Gordonibacter pamelaeae* DSM 19378. When *G. massiliensis* strain Marseille-P2775^T^ was compared with these closely related species, we found values ranging from 70.94% with *Slackia heliotrinireducens* strain DSM 20476 to 85.69% with *G. pamelaeae* strain DSM 19378 (Fig. 4).

## Description of *Gordonibacter massiliensis* sp. nov.

*Gordonibacter massiliensis* (mas.si.li.en'sis. L. fem. adj., from *massiliensis* of Massilia, the Latin name of Marseille where the strain was first cultivated) is a Gram-positive, motile, non-spore-forming and obligate anaerobic coccobacillus. Bacterial cells had a mean diameter of 0.5 μm. Colonies appear beige on blood agar after 48 h of incubation at 37°C in an anaerobic environment. Major cellular fatty acids were 9-octadecenoic acid (41%) and hexadecanoic acid (24%). Catalase is positive but oxidase is negative. Utilization of l-arabinose and d-glucose distinguishes strain Marseille-P2775 from among the closest bacterial species. Also mannose, raffinose, fucose and β-glucosidase are not produced. The strain Marseille-P2775 is isolated from the stool sample of healthy woman living in Saudi Arabia. The G + C content of the genomic DNA is 65.1 mol%.

## Conclusion

Based on phenotypic, genomic and phylogenetic analyses, we formally propose the creation of *Gordonibacter massiliensis* sp. nov., represented here by the strain Marseille-2775. This strain was isolated from a stool sample of a 50-year-old healthy Bedouin woman living in the Jazan region of Saudi Arabia.

## Nucleotide sequence accession numbers

The 16S rRNA and genome sequences were deposited in GenBank under accession numbers LT558845 and LT827128, respectively.

## Deposit in culture collections

Strain Marseille-P2775^T^ was deposited in the Collection de Souches de l’Unité des Rickettsies under the following number: CSUR P2775.

## Conflicts of interest

None to declare.

## Funding sources

This study was supported by the Institut Hospitalo-Universitaire (IHU) Méditerranée Infection, the National Research Agency under the programme *Investissements d'avenir*, reference ANR-10-IAHU-03, the Région Provence Alpes Côte d’Azur and European funding FEDER PRIMI.
